# Pseudo-Thrombotic Microangiopathy Secondary to Vitamin B12 Deficiency

**DOI:** 10.1155/2022/7306070

**Published:** 2022-09-03

**Authors:** Dylan Morrissey, Yuheng Sun, Sarina Koilpillai, Jacqueline Kropf, Steve J. Carlan

**Affiliations:** ^1^Department of Internal Medicine, Orlando Regional Healthcare, Orlando, Florida, USA; ^2^Division of Academic Affairs and Research, Orlando Regional Healthcare, Orlando, Florida, USA

## Abstract

**Background:**

Clinical B_12_ deficiency with hematological or neurological manifestations is rare. An unusual manifestation of B_12_ deficiency is pseudo-thrombotic microangiopathy (TMA), which is characterized by hemolytic anemia, thrombocytopenia, and schistocytosis and only occurs in 2.5% of those with B_12_ deficiency. Pseudo-TMA is misdiagnosed as thrombotic thrombocytopenic purpura (TTP) in 40% of cases, resulting in misguided treatment including plasmapheresis.

**Case:**

A 44-year-old Hispanic presented with 3 weeks of progressively worsening non-radiating chest pain, fatigue, and shortness of breath (SOB). Laboratory findings revealed severe pancytopenia and macrocytosis with a hemoglobin of 5.4 g/dL, mean corpuscular volume of 116.3 fL, and vitamin B_12_ low at 149 pg/mL. She was diagnosed with pseudo-TMA and after starting 1000 micrograms of parenteral vitamin B_12_ injections daily and discontinuing plasmapheresis and steroid administration, she improved.

**Conclusion:**

Failure to recognize pseudo-TMA often results in unnecessary treatment with plasmapheresis and delays appropriate treatment with vitamin B_12_ supplementation. It is therefore extremely important to consider pseudo-TMA as a differential diagnosis in patients that present with hemolytic anemia, thrombocytopenia, and schistocytosis.

## 1. Introduction

Vitamin B_12_ plays an important role in hematopoiesis and neurological function. Clinical manifestations of B_12_ deficiency include megaloblastic anemia, peripheral neuropathy, psychosis, and subacute combined degeneration of the spinal cord. Clinical B_12_ deficiency with hematological or neurological manifestations is rare, but subclinical B_12_ deficiency has an incidence of 2.5–26% in the general population [1]. Pernicious anemia is the most common cause of B_12_ deficiency in the world and is caused by anti-intrinsic factor or anti-parietal cell antibodies, resulting in impaired B_12_ absorption in the terminal ileum [1]. A rare manifestation of B_12_ deficiency is pseudo-thrombotic microangiopathy (TMA), which is characterized by hemolytic anemia, thrombocytopenia, and schistocytosis and only occurs in 2.5% of those with B_12_ deficiency [2]. Due to the rarity of pseudo-TMA, approximately 38.8% of cases are misdiagnosed as thrombotic thrombocytopenic purpura (TTP), resulting in misguided treatment including plasmapheresis [3]. Here, we present a rare cause of pseudo-TMA caused by B_12_ deficiency secondary to undiagnosed pernicious anemia.

## 2. Case Presentation

A 44-year-old Hispanic female with a PMH including insulin dependent diabetes mellitus and essential hypertension presented to the Emergency Department (ED) with 3 weeks of progressively worsening non-radiating chest pain, fatigue, and shortness of breath (SOB). She stated the chest pain was diffuse and that both the chest pain and SOB were worsened with exertion. She also complained of melena, gum bleeding while brushing her teeth, nausea, and loss of taste, but denied fevers, chills, hemoptysis, weight loss, numbness or tingling, diarrhea, or confusion. She reported no tobacco or illicit drug use but did describe drinking 1-2 glasses of wine per week. Her family history was significant for myelodysplastic syndrome in her mother. Initial vital signs were unremarkable aside from being slightly hypertensive to 138/92. Physical examination revealed an obese body habitus, conjunctival pallor, and multiple ecchymoses on her bilateral lower extremities, but did not reveal hepatosplenomegaly or any neurological deficits.

Laboratory findings revealed severe pancytopenia and macrocytosis, hemoglobin (hgb) of 5.4 g/dL (grams per deciliter) (11.6–15 g/dL), hematocrit of 16.4%, mean corpuscular volume of 116.3 fL, (femtoliters) (80–100 (fL), white blood cell count of 3.5 × 10^3^/*µ*L (cells per microliter) (5.0–10.0 × 10^3^/*µ*L), and a platelet count of 28,000/*µ*L (per microliter of blood) (150,000–450,000 platelets/*µ*L. The differential revealed a moderate amount of macrocytes, schistocytes, and teardrop cells. Her troponin I was unremarkable at <0.03 ng/mL (nanograms per milliliter), and her comprehensive metabolic panel revealed normal bilirubin level of 1.0 mg/dL (0.1–1.2 mg/dL milligrams per deciliter) and mildly elevated AST to 52 U/L (units per liter) (10–40 U/L). Her lactate dehydrogenase (LDH) was also elevated at 1,647 IU/L (international units per liter) (140–280 IU/L) and haptoglobin decreased (<30.0 mg/dL) (41–165 mg/dL), and peripheral blood smear showed macrocytic normochromic anemia with severe anisopoikilocytosis, frequent schistocytes, and occasional tear drop cells, and no hypersegmented neutrophils were noted. Computed tomography (CT) scan of the chest, abdomen, and pelvis revealed no splenomegaly or any evidence of neoplastic disease. Disseminated intravascular coagulation profile revealed a normal prothrombin time test (PT) of 11.2 seconds, normal fibrinogen of 349 mg/dL (200–400 mg/dL), and a slightly reduced activated partial thromboplastin time (aPTT) of 24.6 seconds. The patient was transfused 2 units of packed red blood cells (pRBCs) with improvement in hgb to 7.3 g/dL, and hematology was promptly consulted due to concern for thrombotic thrombocytopenic purpura (TTP). The patient was started on 100 mg prednisone due to a concern for possible autoimmune hemolytic anemia process and was started on daily plasmapheresis on day 1 of hospitalization.

During the first 2 days of hospitalization, further testing revealed ADAMTS13 level within normal limits at 85% activity, antinuclear antibody (ANA) screen negative, and flow cytometry revealed no paroxysmal nocturnal hemoglobinuria (PNH) clone. ADAMTS13 is a key metalloproteinase enzyme that functions in regulating clotting. Vitamin B_12_ and folate levels revealed low B_12_ (149 pg/mL) (picograms per milliliter) (190–950 (pg/mL) and a normal folate level at 17.5 ng/mL (nanograms per milliliter) (3–13 (ng/mL). She had no improvement in her pancytopenia or symptoms with 2 days of plasmapheresis, and decision was made to initiate 1000 mcg (micrograms) of parenteral cyanocobalamin (vitamin B_12_) injections daily and stop plasmapheresis and steroid administration. The patient's blood counts in all cell lines began to improve after administration of vitamin B_12_, and her symptoms of chest pain and dyspnea on exertion improved. To evaluate the cause of her B_12_ deficiency, anti-parietal cell antibodies and anti-intrinsic factor antibodies were collected, and both returned positive. Serum methylmalonic acid and homocysteine levels were elevated at 23 umol/mL (micromoles per milliliter) (normal range: 0.00-0.40 umol/mL) and >50 umol/L (normal range: 5-15 umol/L), respectively. Her final diagnosis was pseudo-thrombotic microangiopathic anemia (pseudo-TMA) secondary to pernicious anemia, and the patient was discharged home with monthly B_12_ injections and follow-up in the hematology clinic in 2 months.

## 3. Discussion

Vitamin B_12_ (cobalamin) serves an essential physiological role as a cofactor in DNA/RNA synthesis and homocysteine metabolism. B_12_ deficiency can lead to ineffective hematopoiesis and demyelination, which classically presents with pancytopenia, megaloblastic anemia, hypersegmented neutrophils, and dorsal column dysfunction. In adults, the most common etiology is autoimmune pernicious anemia, in which case autoantibodies against the intrinsic factor-B_12_ complex impairs absorption. Other causes include inadequate dietary intake, malabsorptive causes, and genetic etiologies. Cobalamin metabolism defect (*Cbl* C) is an autosomal recessive disorder of intracellular cobalamin processing caused by mutations in *Mmachc* gene. Generally these inherited defects are diagnosed early in life, and, in fact, many newborn screening programs can detect Cbl C [4].

In a rare 10% of cases, cobalamin deficiency can present with hemolysis. The pathophysiology is still poorly understood, but two proposed mechanisms involve intermedullary hemolysis and hyperhomocysteinemia. Intermedullary hemolysis occurs due to fragile RBC membranes, which cause them to shear before they reach the peripheral circulation. Elevated homocysteine levels have been shown to downregulate the activity of glutathione peroxidase, leading to the accumulation of reactive oxygen species that damage the endothelium and further lyse the already fragile RBCs. When hemolytic anemia is associated with thrombocytopenia and schistocytes in the setting of B_12_ deficiency, the triad is termed pseudo-thrombotic microangiopathy (PTMA). This is an extremely rare presentation of B12 deficiency that is only seen in 2.5% of cases.

Importantly, the hemolysis seen in cobalamin deficiency occurs in the absence of intravascular platelet aggregates or microthrombi, which is the defining hallmark of a group of disorders termed thrombotic microangiopathies. The most common causes of thrombotic microangiopathies include diffuse intravascular coagulopathy (DIC), thrombotic thrombocytopenic purpura (TTP), and hemolytic uremia syndrome (HUS). TTP is of particular importance as it shares the same triad of laboratory findings as PTMA. Fever, neurological findings, and renal damage may also be present in TTP but are not necessarily required for diagnosis. TTP is a medical emergency that is almost always fatal without prompt initiation of treatment. Therefore, up to 39% of PTMA patients are initially misdiagnosed with TTP. This occurred in our clinical case, as our patient was started on empiric plasmapheresis until ADAMTS came back at a normal value and we considered other differentials.

Studies comparing PTMA with TTP have helped elucidate additional differences between the two diagnoses. PTMA is associated with very elevated LDH levels, mild bilirubin elevations, high MCV, low reticulocyte counts, and a Coombs-positive pernicious anemia in a majority of cases. Markedly elevated LDH occurs from hemolysis of nucleated RBCs and would therefore not be expected in TTP as the mature RBCs being lysed are anucleate. Similarly, erythroid progenitors produce smaller amounts of hemoglobin and cause mild bilirubin elevation. Macrocytosis coupled with low reticulocytes is due to ineffective erythropoiesis from B12 deficiency. This combination in particular is suggestive of PTMA as opposed to TTP. Finally, TTP would present with a Coombs-negative anemia as it is not an autoimmune etiology. When looking back at our clinical case, all of these subtle signatures can be seen.

Once the diagnosis of PTMA is made, treatment is B12 supplementation. Most patients respond to therapy with resolution of lab abnormalities and clinical symptoms.

## 4. Conclusion

Pseudo-thrombotic microangiopathy is an extremely rare clinical manifestation of vitamin B_12_ deficiency that results from shearing of red blood cells (RBCs) in the absence of microthrombi due to inherent RBC fragility. In contrast to thrombotic microangiopathies, reticulocyte count is low in pseudo-TMA due to limited B_12_ reserves [5]. Given the rarity of the disease, pseudo-TMA is often not considered early in the workup of a suspected microangiopathic hemolytic anemia [6]. Failure to recognize pseudo-TMA often results in unnecessary treatment with plasmapheresis and delays appropriate treatment with vitamin B_12_ supplementation. It is therefore extremely important to consider pseudo-TMA as a differential diagnosis in patients that present with hemolytic anemia, thrombocytopenia, and schistocytosis.

## Data Availability

The data generated or analyzed in this study are included within this article.

## References

[1] Green R., Allen L. H., Bjørke-Monsen A. L. (2017). Vitamin B_12_ deficiency. *Nature Reviews Disease Primers*.

[2] Tran P. N., Tran M.-Ha (2018). Cobalamin deficiency presenting with thrombotic microangiopathy (TMA) features: a systematic review. *Transfusion and Apheresis Science*.

[3] Tun A. M., Myint Z. W., Rojas Hernandez E. G. (2017). Kyaw Zin Thein, and Thein Hlaing Oo. Vitamin B_12_ deficiency-related pseudo-thrombotic microangiopathy might be misdiagnosed and treated with plasma product therapy: review of the literature and analysis of the reported cases. *Blood*.

[4] Weisfeld-Adams J. D., Bender H. A., Miley-Akerstedt A. (2013). Neurologic and neurodevelopmental phenotypes in young children with early-treated combined methylmalonic acidemia and homocystinuria, cobalamin C type. *Molecular Genetics and Metabolism*.

[5] Rao S., Colon Hidalgo D., Doria Medina Sanchez J. A., Navarrete D., Berg S. (2020). B_12_ Cobalamin deficiency masquerading as pseudo-thrombotic microangiopathy. *Cureus*.

[6] Hassouneh R., Shen S., Lee O., Hart R. A., Rhea L. P., Fadden P. (2021). Severe vitamin B_12_ deficiency mimicking microangiopathic hemolytic anemia. *Journal of Hematology*.

